# Inference of Selection Based on Temporal Genetic Differentiation in the Study of Highly Polymorphic Multigene Families

**DOI:** 10.1371/journal.pone.0042119

**Published:** 2012-08-10

**Authors:** Mark McMullan, Cock van Oosterhout

**Affiliations:** School of Environmental Sciences, University of East Anglia, Norwich Research Park, Norwich, United Kingdom; University of London, St George's, United Kingdom

## Abstract

The co-evolutionary arms race between host immune genes and parasite virulence genes is known as Red Queen dynamics. Temporal fluctuations in allele frequencies, or the ‘turnover’ of alleles at immune genes, are concordant with predictions of the Red Queen hypothesis. Such observations are often taken as evidence of host-parasite co-evolution. Here, we use computer simulations of the Major Histocompatibility Complex (MHC) of guppies (*Poecilia reticulata*) to study the turnover rate of alleles (temporal genetic differentiation, *G'_ST_*). Temporal fluctuations in MHC allele frequencies can be ≥≤order of magnitude larger than changes observed at neutral loci. Although such large fluctuations in the MHC are consistent with Red Queen dynamics, simulations show that other demographic and population genetic processes can account for this observation, these include: (1) overdominant selection, (2) fluctuating population size within a metapopulation, and (3) the number of novel MHC alleles introduced by immigrants when there are multiple duplicated genes. Synergy between these forces combined with migration rate and the effective population size can drive the rapid turnover in MHC alleles. We posit that rapid allelic turnover is an inherent property of highly polymorphic multigene families and that it cannot be taken as evidence of Red Queen dynamics. Furthermore, combining temporal samples in spatial *F_ST_* outlier analysis may obscure the signal of selection.

## Introduction

Currently, a major challenge in evolutionary biology is to understand why the level of genetic variation and differentiation varies among genes that occur within the same genome [1]. Assuming that the effects of random genetic drift and migration are similar across the genome, loci with significantly high or low levels of genetic differentiation, outlier loci, are likely to be affected by other evolutionary forces [2]–[4]. Many high throughput sequencing studies aim to identify genomic regions under selection [5]. Whereas purifying and directional selection tend to erode genetic variation below the level expected from neutral evolution, balancing selection helps to maintain polymorphism [6], [7], though its effect is still mediated by the effective population size [8], [9]. The signal of non-neutral evolution can be inferred from *F_ST_*-outlier analyses [10], [11], in which loci with a significantly reduced level of spatial genetic differentiation are considered to be under balancing selection [12].

Balancing selection has been particularly well studied at the genes of the Major Histocompatibility Complex (MHC) [13]. These genes play a central role in the vertebrate immune system, and given that both their molecular structure and function are conserved across vertebrates [14], the MHC has become a paradigm to study selection in non-model organisms in the wild [15]. In most outbreeding populations, the MHC is highly polymorphic and this diversity is thought to be maintained by some form of balancing selection by parasites [13], [15].

There are several models of balancing selection that all increase the gene diversity (heterozygosity) within loci relative to neutrally evolving genes [16]–[18]. The impact of balancing selection on genetic differentiation can differ fundamentally depending on the particular mode of balancing selection. Fluctuating selection is predicted to favour different MHC alleles at different times, and it can therefore lead to increased levels of genetic differentiation [19]. Overdominant and negative frequency dependent selection, however, tend to reduce genetic differentiation [13], [20]–[22]. The mode of selection can thus be inferred based on the level of spatial and temporal genetic differentiation, which can help to elucidate the evolutionary processes acting on the studied gene or gene family.

The co-evolutionary arms race between parasite virulence genes and host immune genes is known as Red Queen dynamics [23]. To empirically demonstrate Red Queen dynamics is particularly challenging because it requires a time-series analysis of both host and parasite genotypes. Hence, only few studies provide evidence of co-evolutionary dynamics [24]–[26]. Instead, most studies simply demonstrate a pattern of variation in allele and genotype frequency that is consistent with co-evolution [27]. In those cases, the rapid turnover rate of alleles is often taken as evidence of Red Queen dynamics.

Here we explore the impact of evolutionary forces and demography on the spatial and temporal dynamics of the MHC. We build an individual based model to simulate the interactions between random genetic drift, gene flow and selection with the aim to further our understanding of what can be inferred from temporal allele frequency fluctuations in highly polymorphic multigene families.

## Methods and Models

An individual based model was written to study how the change in MHC allele frequencies over time (temporal genetic differentiation, or ‘turnover’), is affected by balancing selection, gene flow, effective population size, population size fluctuations and the level of polymorphism at the locus. We study allele frequency changes across 18 generations so that we can compare our simulations to empirical data, i.e. temporal samples of guppy (*Poecilia reticulata*) populations of the Caroni Drainage in Trinidad [28]. To quantify turnover rate we use *G'_ST_*, a standardised measure of genetic differentiation that accounts for allele frequency variation at highly polymorphic loci [29].

We parameterise our computer model, basing it on the demography and population genetics of guppies in the Caroni Drainage in Trinidad [6], [30]. Guppies of the Caroni drainage occur in a source–sink metapopulation [30]. Downstream migration occurs during the wet season rains when the rivers are in spate [31]. After seasonal floods, the census population size of guppies in the downstream Caura catchment is estimated to be tens to hundreds of millions. In the dry season, guppies migrate upstream and these seasonal migration events create a highly dynamic metapopulation structure [30].

This metapopulation structure was modelled simulating an upstream and a downstream river population that were connected by gene flow. We simulated the MN (an upstream population with *N_e_* = 100), the LA (downstream, *N_e_* = 2400) and the infinitely large “super-sink" population (the Caroni catchment) [6], [30]. The level of admixture ranged from 5–21% (mean 12%) for the downstream populations of guppies [30]. Unless noted otherwise, population demography was affected by a flushing event each year to simulate annual rains [31]. Flushing events were followed by upstream migration [*Nm* = 1, see 28] and subsequent population expansion. Genetic variation at the MHC was simulated using the symmetric overdominance model (heterozygote advantage) [32], with selection coefficient (*s* = 0–0.5), the number of MHC alleles in the Caroni drainage (*k* = 5–80), effective population size (*N_e_* = 30–1000), migration rate (*Nm* = 0.2–10.0) and the time of year that migration took place (continual migration or seasonal migration). Given the relatively small number of simulated generations (*μN_e_t*«1) and the relatively high migration rate (*Nm*≥1 [28]), the model did not include mutation (*μ* = 0). There is free recombination (*c* = 0.5) and fitness is multiplicative across loci.

Guppies are thought to possess (up to) three MHC class II*B* gene loci which are highly polymorphic [6], [33], [34]. By combining three temporal datasets of the MHC of guppies in the Caroni Drainage [6], [28], [33], we estimate that there are approximately 85 MHC alleles in total in the Drainage as a whole, although individual populations possess only a fraction of these alleles [33]. The locus-specificity of these alleles remains unknown, and given the evidence of inter-locus recombination [34], [35], the simulations assumed each allele can occur at any of these three loci.

The temporal *G'*_ST_ was calculated for the upstream population sampled in two sampling periods separated by *t* = 18 generations. Simulations were started with genotypes sampled at random from the source population (containing *k* MHC alleles). Data were recorded after a burn-in period of *t* = 1000 generations, which was sufficient to reach an initial selection-drift equilibrium.

## Results

### Factors influencing the temporal differentiation of the MHC and microsatellites

We delineated the effects of the strength of (overdominant) selection, the level of polymorphism, migration rate, effective population size, and population size fluctuations on the temporal *G'*_ST_.

#### Selection coefficient

**Figure 1:**
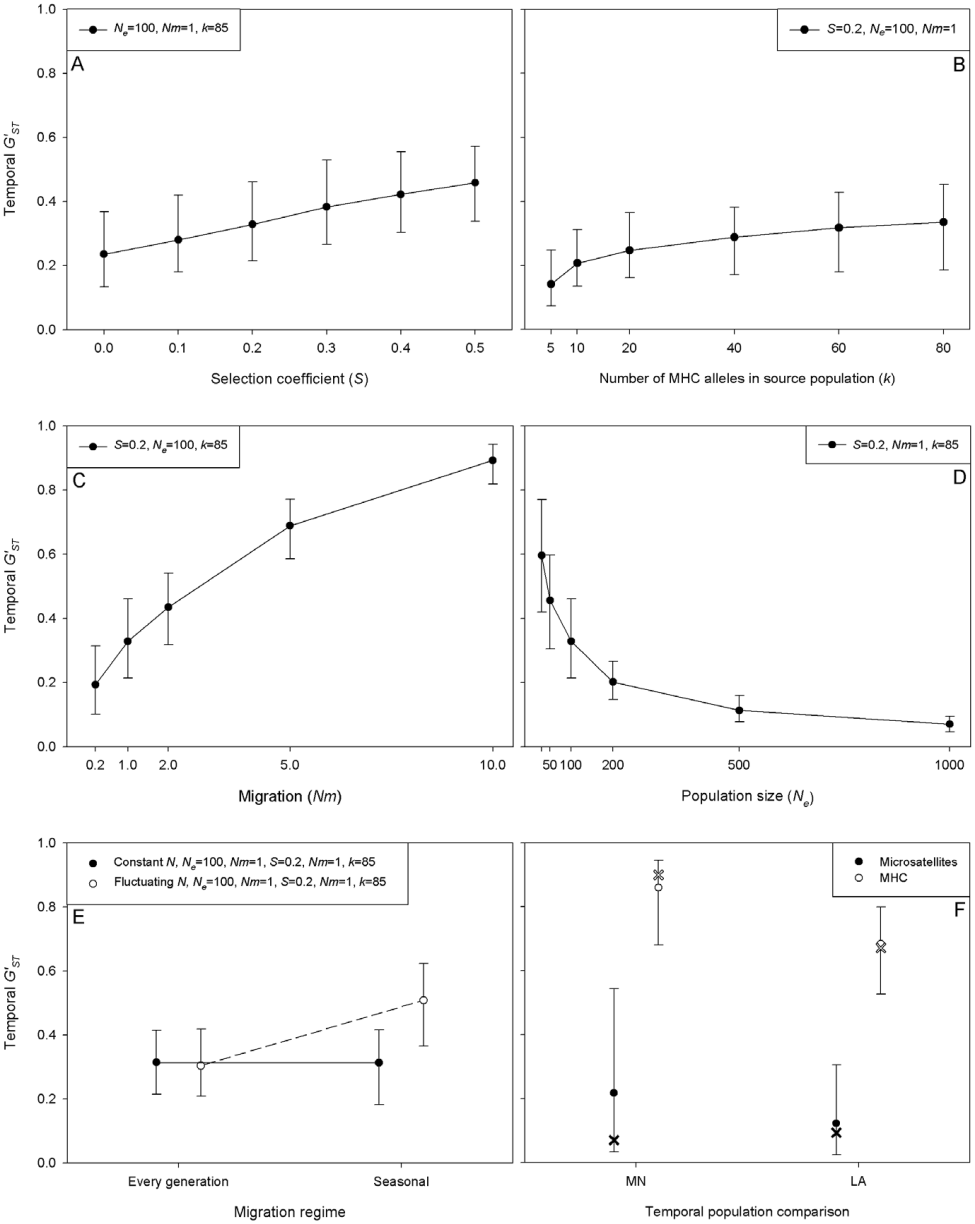
Temporal genetic differentiation (*G'_ST_*) in simulated populations under various demographic and population genetic scenarios. Unless stated otherwise, populations are of constant effective population size *N_e_* = 100, gene flow *Nm* = 1 from a source population with 85 distinct MHC alleles (*k* = 85), with alleles being maintained by symmetric overdominant selection with *s* = 0.2. Error bars indicate 5–95% confidence interval. (A) The coefficient of selection ranges between 0≤*s*≤0.5. (B) The number of MHC alleles in the source metapopulation ranges between 5≤*k*≤80 distinct alleles. (C) Upstream migration rate ranges between 0.2≤*Nm*≤10.0 individuals per generation. The dashed grey line represents inferred value for 3*Nm* (D) The effective population size ranges between 30≤*N_e_*≤1000. (E) Migration (*Nm* = 1) is either every generation or seasonal (every third generation), into a constant (solid circles) or fluctuating (open circles) population of harmonic mean size *N_H_* = 100. (F) The temporal genetic differentiation within the MN and LA populations over time (2001–2007) at microsatellite (closed symbols) and MHC loci (open symbols). These simulations use fixed parameter estimates from previous runs (panels A–E) combined with annually fluctuating census population size migration. Simulated (mean *G'_ST_* (5–95% CI)) and the mean observed genetic differentiation (*G'_ST_*) are represented by circles and crosses, respectively (see Tables S1, S2, S3 for data).

The temporal *G'_ST_* increased with the coefficient of selection (Fig. 1A, Table S1A). The initially rare immigrant alleles were selectively favoured because they are more likely to be in a heterozygous state and therefore, they tended to increase rapidly in frequency. Assuming that the number of alleles that can be maintained in the genepool is already at equilibrium, the invasion of a novel immigrant allele increases the likelihood that an established allele will be lost by drift. Overdominant selection (as well as negative frequency dependent selection) therefore increases the allelic turnover rate. Assuming all else is equal, an MHC locus with selection coefficient *s* = 0.2 will show a temporal *G'_ST_* that is 28.6% higher than a neutral microsatellite locus in our guppy metapopulation (see Table S1A).

#### Number of MHC alleles

The temporal *G'_ST_* increased with the level of polymorphism in the metapopulation (Fig. 1B, Table S1B). If there were more distinct MHC alleles, immigrants were more likely to introduce novel MHC alleles. The novel immigrant alleles can potentially replace the resident alleles, thereby increasing the temporal *G'_ST_*. Compared to a locus with 20 alleles (typical for a guppy microsatellite locus), a locus that has 80 MHC alleles in the metapopulation will show a 26.6% higher rate of temporal *G'_ST_*. Note that within a given natural metapopulation, the number of alleles is not independent but the product of the interaction between the effective (meta) populations size, the migration rate, and the level of balancing selection [22].

#### Migration

The impact of migration on the temporal *G'_ST_* was particularly strong (Fig. 1C, Table S1C). This observation is relevant for MHC and other genes with copy number variation. In guppies, there are three duplicated class II*B* genes and given copy number variation [34], the effective rate of gene flow is three times higher than that of a microsatellite. Consequently, the level of temporal *G'_ST_* is 36.4% higher than that of a microsatellite locus.

#### Population size

The allele frequency spectra in large populations were relatively stable and less affected by migration than small gene pools. Consequently, the temporal *G'_ST_* was highest in the simulations of populations with small *N_e_* (Fig. 1D, Table S1D). Given that the *N_e_* of guppy populations analysed are relatively small [30], this will result in large temporal allele frequency fluctuations. This will affect, however, the temporal *G'_ST_* of both the MHC and the microsatellites to a similar extent.

#### Population size fluctuations

Figure 1E shows that when the migration rate was constant, population size fluctuations did not affect the temporal *G'_ST_*. However, population size fluctuations did increase the level of temporal genetic differentiation when migration occurs after a population crash (Fig. 1E, Table S2). In the guppy metapopulation, MHC alleles that are introduced by immigrants after the wet season floods are likely to become established when the population rapidly expands during a population-boom following the wet season population-crash. Under the given parameter settings, population size fluctuations increase the level of temporal genetic divergence to a similar extent as an increase in the selection coefficient from *S* = 0 to 0.4.

### What explains the difference among neutral and MHC temporal differentiation?

Finally, we used the individual based model to examine the discrepancy in *G'_ST_* between MHC and microsatellites observed by McMullan [28]. In that study, the temporal MHC variation was an order of magnitude higher than that of microsatellites (see Fig. 1F), a finding that would usually be interpreted as evidence for fluctuating selection (i.e. Red Queen dynamics) [33]. In these simulations we assumed a selection coefficient of *s* = 0.2 for the MHC [6], [31] and *s* = 0 for the microsatellites. Levels of polymorphism and demographic parameters were taken from Barson et al. [30]. Figure 1F shows that the observed high level of temporal *G'_ST_* of the MHC and the relatively low level of temporal *G'_ST_* of the microsatellites are both consistent with the simulations (see also, Table S3). The synergistic interaction between overdominant selection, the high level of polymorphism, a high rate of gene flow due to copy number variation, and fluctuations in population size, are elevating the temporal genetic differentiation of the MHC to a level above that observed at neutral microsatellite loci.

## Discussion

We assess how overdominant selection affects temporal divergence in the MHC, taking into account the impact of population demography and fluctuations, as well as the particular properties of this multigene family (i.e. copy number variation and a large number of distinct alleles). We used an individual based model of overdominant selection within a dynamic metapopulation system based on guppy populations of the Caroni Drainage in Trinidad. We showed that overdominant selection tends to increase temporal genetic differentiation in MHC. This observation could mistakenly be interpreted as fluctuating selection and taken as evidence for Red Queen dynamic co-evolution. Our model showed, however, that the synergistic interaction between the evolutionary forces and demographic processes in this metapopulation can explain the rapid turnover of MHC alleles, without a changing parasite fauna. This may explain why some studies report a high turnover rate of MHC alleles with apparently modest changes in parasite fauna (see Table 1). In addition, we show that the impact of balancing selection on genetic differentiation differs depending on whether the signal is analysed across a spatial or temporal scale, which has important implications for sampling design.

**Table 1:** Summary of Temporal MHC studies including population demographic parameters important for generating temporal variation.

Organism	Locus	Fluctuating *s*	Fluctuating *N_c_*	Parasite screen	Populations	Alleles	Ref
Great read warblers	*MHC I*	Yes	No	No	1	67	[50]
Soay Sheep	*MHC II*	Yes	Yes	No	4	8	[51]
Water vole (France)	*MHC II*	Yes	Yes	No	7	16	[52]
Brown trout	*TAP2A*	Yes	Yes	No	11	14	[53]
Water vole (Scotland)	*MHC II*	No	Yes	No	3	6	[38]
Brown hares	*MHC II*	No	No	Yes	1	10	[54]
Guppy	*MHC II*	Yes	Yes	Yes	9	66	[33]
Greater prairie-chickens	*MHC II*	No	Yes	No	1	24	[8]

‘Fluctuating *s*’ refers to studies which concluded that fluctuating selection may have increased temporal differentiation of immune genes relative to that of neutral markers. Studies that provided either direct evidence for changes in population size, or temporally unstable population structure, were considered to have a fluctuating census population size (‘Fluctuating *N_c_*’). ‘Populations’ indicates the number of sampled populations and ‘Alleles’ indicates the total observed number of MHC alleles in the sampled metapopulation.

### Population genetic factors and temporal genetic differentiation

Our simulations show that the temporal genetic differentiation increased markedly when migration occurred seasonally in small populations that fluctuated in size. Under these scenarios, novel immigrant alleles replaced the resident alleles that were lost during the bottleneck. This is concordant with Ejsmond and Radwan [9] who showed that population bottlenecks can be more pronounced for the MHC than for alleles at a neutral loci.

Temporal genetic differentiation is strongly influenced by population genetic features specific to each gene or gene family, including the selection coefficient and the level of genetic polymorphism. The temporal differentiation increased with the coefficient of balancing selection (symmetric overdominance), the level of genetic polymorphism in the metapopulation and the effective rate of gene flow. These population genetic parameters differ fundamentally between (neutral) microsatellite loci and MHC immune genes. Previous work has shown that balancing selection increases the turnover rate of alleles relative to that of neutral genes [18], and here we analyse the effects of the level of genetic polymorphism present at multiple, duplicated genes.

MHC genes are often highly polymorphic, with for example over 1000 alleles found at the human MHC (*DRB1*) [36]. The computer simulations show that a high level of genetic polymorphism further accelerates the rate of genetic differentiation with time. Logically, the probability that an immigrant introduces a novel allele increases with the total number of distinct alleles. Simulating a metapopulation of guppies, we showed that, due to its higher level of polymorphism, the temporal genetic differentiation of the MHC is expected to be 26.6% higher than that of a typical microsatellite locus, irrespective of any other differences between these genes. This has important consequences for research conducted on populations within a metapopulation because a higher effective migration rate of alleles at balanced loci [20], [22] will increase temporal genetic differentiation.

Furthermore, the effective rate of gene flow differs between MHC and microsatellites. In multigene families with many duplicated genes, each migrant can introduce more ‘alleles’ into the recipient gene pool. Guppies have three duplicated MHC class II*B* genes, and hence, the effective rate of gene flow is potentially three times greater for MHC than for microsatellites. This is relevant for conservation biology, given that MHC variation appears to be more prone to genetic drift during bottlenecks than microsatellite variation [8], [37]. Migration can negate the genetic depletion caused by population fragmentation, particularly to replenish variation at multigene families such as the MHC.

Our individual based model can explain stark differences in temporal genetic differentiation between the MHC and microsatellites using overdominant selection and the synergistic interaction with demography and can account for a turnover of MHC alleles. We find that population demographic parameters such as the migration rate and effective population size have a surprising influence on the rate of MHC allele turnover in comparison to selection. Several studies have reported large temporal changes in MHC allele frequencies (see Table 1), but there are notable exceptions. For example, Oliver *et al.* [36] observed that the temporal genetic differentiation was low. Water vole populations studied by Oliver *et al.* [38] possess only few MHC alleles at a single MHC class II *DRB* locus (Table 1), which reduces the temporal genetic differentiation, a finding that is consistent with the present simulations. Interestingly, allele frequency fluctuations occur between generations at the self-incompatibility locus (S-locus) in plants [39], [40]. The S-locus generally has high levels of polymorphism and temporal variation in allele frequencies is thought to be caused by negative frequency dependent selection favouring rare alleles in successive generations [39], [40]. In our model, novel MHC alleles are favoured during immigration events because of the advantage they convey as heterozygotes. The present study highlights that we can only fully understand the evolutionary dynamics of the MHC by incorporating all relevant population genetic aspects, i.e. the level of genetic polymorphism, the number of duplicated genes and hence, the effective rate of gene flow, as well as the strength of balancing selection.

### Red Queen dynamics and temporal genetic differentiation

Our computer study demonstrates that an apparent signal of Red Queen dynamics can be explained by the population genetic characteristics of this multigene family and the demography of metapopulations. However, our results should not be interpreted as evidence that Red Queen dynamics do not occur. The empirical evidence supporting host-parasite co-evolution is strong [24], [25], albeit often indirect [7], [41]. Many studies have directly correlated the infection rate of hosts to their MHC genotype or to particular MHC alleles [42]–[46], but there are several reasons as to why demonstrating the causal link between the immunological function of the MHC and the rich parasite biodiversity remains challenging [47], [48].

Other selective processes may also act on the MHC and weaken the direct association between parasites and immunogenetic variation. For example, sexual selection may also be operating on the MHC variation. MHC odour cues appear to play a role in mate choice, with preference given to MHC-dissimilar mates in several species [49]. Similarly, selection against a sheltered load of recessive deleterious mutations could also maintain a balanced polymorphism, even in the absence of parasite-mediated selection (*cf.* ABC evolution [18], [34]).

### Implications for a locus evolving under balancing selection

Previous work has shown that overdominance and negative frequency dependent selection tend to homogenise allele frequencies and reduce spatial genetic differentiation [20], [21]. The current study shows, however, that the opposite may be true of populations sampled at different times, and therefore, if samples are taken across different generations, the level of genetic differentiation would appear (artificially) inflated. Combining population samples across generations could confound the effect of balancing selection on the theoretically predicted *F_ST_*.

### Conclusions

This study shows that the effect of migration can differ fundamentally between genes within the same genome. Migration can result in large temporal genetic divergence, particularly at highly polymorphic genes in multigene families with copy number variation. Large changes in allele frequencies do not necessarily imply temporal variation in natural selection by parasites and so we feel that such conclusions must be bolstered by parasite prevalence data. This study highlights the importance of incorporating the interactions between the evolutionary forces with population demography, which requires an in depth understanding of both the genetics and ecology of the study species.

## Supporting Information

Table S1 **Temporal genetic differentiation (** ***G'_ST_*** **(5–95%CI)) over 18 generations calculated for a simulated upstream population.** Each data panel (A–D) represents a demographic scenario and corresponds to panels used in Figure 1 which include (A) the selection coefficient, (B) the number of MHC alleles in the metapopulation, (C) the migration rate, and (D) the effective population size. *G'_ST_* for 3*Nm* (panel C) is inferred from the figure.
(DOC)

Table S2 **Temporal genetic differentiation (** ***G'_ST_*** **(5–95%CI)) over 18 generations calculated for a simulated upstream population (refer to** **Fig. 1E** **).** The census population size was either constant or fluctuating and immigration occurred either every generation (three times per year) or once per year (seasonal).
(DOC)

Table S3 **Temporal genetic differentiation (** ***G'_ST_*** **(5–95%CI)) over 18 generations calculated separately for microsatellite and MHC loci for an upstream (MN) and a downstream (LA) population (refer to** **Fig. 1F** **).** Observed temporal genetic differentiation is encompassed within simulated results from the present study (see Fig. 1F).
(DOC)
