# Supplementary material for: Inference of Selection Based on Temporal Genetic Differentiation in the Study of Highly Polymorphic Multigene Families
Source: PLoS One. 2012 Aug 10;7(8):e42119. doi: 10.1371/journal.pone.0042119 (PMC3416836; doi:10.1371/journal.pone.0042119)
Supplement: Table S1 — Temporal genetic differentiation ( G'ST (5–95%CI)) over 18 generations calculated for a simulated upstream population. Each data panel (A–D) represents a demographic scenario and corresponds to panels used in Figure 1 which include (A) the selection coefficient, (B) the number of MHC alleles in the metapopulation, (C) the migration rate, and (D) the effective population size. G'ST for 3Nm (panel C) is inferred from the figure. (DOC) [file pone.0042119.s001.doc]

Table S1. Temporal genetic differentiation (*G'_ST_* (5-95%CI)) over 18 generations calculated for a simulated upstream population. Each data panel (A-D) represents a demographic scenario and corresponds to panels used in Figure 1 which include (A) the selection coefficient, (B) the number of MHC alleles in the metapopulation, (C) the migration rate, and (D) the effective population size. *G’_ST_* for 3*Nm* (panel C) is inferred from the figure.

| **A)** | ***s*** | ***G'_ST_*** |  | **B)** | **No. *A*** | ***G'_ST_*** |  |
| --- | --- | --- | --- | --- | --- | --- | --- |
|  | 0 | 0.235 | (0.134-0.368) |  | 5 | 0.141 | (0.073-0.248) |
|  | 0.1 | 0.280 | (0.180-0.420) |  | 10 | 0.206 | (0.135-0.311) |
|  | 0.2 | 0.329 | (0.214-0.461) |  | 20 | 0.246 | (0.161-0.365) |
|  | 0.3 | 0.383 | (0.266-0.529) |  | 40 | 0.288 | (0.171-0.380) |
|  | 0.4 | 0.422 | (0.304-0.555) |  | 60 | 0.317 | (0.179-0.428) |
|  | 0.5 | 0.458 | (0.338-0.573) |  | 80 | 0.335 | (0.185-0.453) |
| **C)** | ***Nm*** | ***G'_ST_*** |  | **D)** | ***N_e_*** | ***G'_ST_*** |  |
|  | 0.2 | 0.193 | (0.101-0.315) |  | 30 | 0.596 | (0.420-0.771) |
|  | 1 | 0.329 | (0.214-0.461) |  | 50 | 0.457 | (0.305-0.597) |
|  | 2 | 0.434 | (0.318-0.542) |  | 100 | 0.329 | (0.214-0.461) |
|  | 3 | 0.517 |  |  | 200 | 0.202 | (0.146-0.267) |
|  | 5 | 0.689 | (0.586-0.771) |  | 500 | 0.113 | (0.078-0.159) |
|  | 10 | 0.893 | (0.819-0.943) |  | 1000 | 0.070 | (0.046-0.095) |
