# Supplementary material for: Inference of Selection Based on Temporal Genetic Differentiation in the Study of Highly Polymorphic Multigene Families
Source: PLoS One. 2012 Aug 10;7(8):e42119. doi: 10.1371/journal.pone.0042119 (PMC3416836; doi:10.1371/journal.pone.0042119)
Supplement: Table S2 — Temporal genetic differentiation ( G'ST (5–95%CI)) over 18 generations calculated for a simulated upstream population (refer to Fig. 1E ). The census population size was either constant or fluctuating and immigration occurred either every generation (three times per year) or once per year (seasonal). (DOC) [file pone.0042119.s002.doc]

Table S2. Temporal genetic differentiation (*G'_ST_* (5-95%CI)) over 18 generations calculated for a simulated upstream population (refer to Fig. 1E). The census population size was either constant or fluctuating and immigration occurred either every generation (three times per year) or once per year (seasonal).

|  | ***G’_ST_* Constant *N* (*N_e_*=100)** | | ***G’_ST_* Fluctuating *N* (*N_e_*=100)** | |
| --- | --- | --- | --- | --- |
| **Every Generation** | 0.315 | (0.215-0.415) | 0.304 | (0.208-0.418) |
| **Seasonal** | 0.313 | (0.182-0.416) | 0.508 | (0.365-0.623) |
