# Supplementary material for: Inference of Selection Based on Temporal Genetic Differentiation in the Study of Highly Polymorphic Multigene Families
Source: PLoS One. 2012 Aug 10;7(8):e42119. doi: 10.1371/journal.pone.0042119 (PMC3416836; doi:10.1371/journal.pone.0042119)
Supplement: Table S3 — Temporal genetic differentiation ( G'ST (5–95%CI)) over 18 generations calculated separately for microsatellite and MHC loci for an upstream (MN) and a downstream (LA) population (refer to Fig. 1F ). Observed temporal genetic differentiation is encompassed within simulated results from the present study (see Fig. 1F). (DOC) [file pone.0042119.s003.doc]

Table S3. Temporal genetic differentiation (*G'_ST_* (5-95%CI)) over 18 generations calculated separately for microsatellite and MHC loci for an upstream (MN) and a downstream (LA) population (refer to Fig. 1F). Observed temporal genetic differentiation is encompassed within simulated results from the present study (see Fig. 1F).

|  | **Microsatellites** | | | **MHC** | | |
| --- | --- | --- | --- | --- | --- | --- |
|  | **Obs *G'_ST_*** | **Sim *G'_ST_*** |  | **Obs *G'_ST_*** | **Sim *G'_ST_*** |  |
| **MN** | 0.071 | 0.218 | (0.034-0.545) | 0.900 | 0.860 | (0.681-0.947) |
| **LA** | 0.094 | 0.123 | (0.025-0.306) | 0.672 | 0.684 | (0.527-0.799) |
